# Effect of topical microporous polysaccharide hemospheres on the duration and amount of fluid drainage following mastectomy: a prospective randomized clinical trial

**DOI:** 10.1186/s12885-019-5293-1

**Published:** 2019-01-23

**Authors:** Lorena P. Suarez-Kelly, W. Hampton Pasley, Eric J. Clayton, Stephen P. Povoski, William E. Carson, Ray Rudolph

**Affiliations:** 1 Memorial University Medical Center, 4700 Waters Ave, Savannah, GA 31404 USA; 2 The Arthur G. James Comprehensive Cancer Center and Solove Research Institute, The Ohio State University, 424 Wiseman Hall, 410 W. 12th Ave, Columbus, OH 43210 USA

**Keywords:** Breast cancer, Mastectomy, Microporous polysaccharide hemospheres

## Abstract

**Background:**

Seroma formation is the most common complication after mastectomy and places patients at risk of associated morbidities. Microporous polysaccharide hemospheres (MPH) consists of hydrophilic, plant based, polysaccharide particles and is currently used as an absorbable hemostatic agent. An animal model evaluating MPH and seroma formation after mastectomy with axillary lymph node dissection showed a significant decrease in seroma volume. Study aim was to evaluate topical MPH on the risk of post-mastectomy seroma formation as measured by total drain output and total drain days.

**Methods:**

Prospective randomized single-blinded clinical trial of patients undergoing mastectomy for the treatment of breast cancer. MPH was applied to the surgical site in the study group and no application in the control group.

**Results:**

Fifty patients were enrolled; eight were excluded due to missing data. Forty-two patients were evaluated, control (*n* = 21) vs. MPH (*n* = 21). No difference was identified between the two groups regarding demographics, tumor stage, total drain days, total drain output, number of clinic visits, or complication rates. On a subset analysis, body mass index (BMI) greater than 30 was identified as an independent risk factor for high drain output. Post hoc analyses of MPH controlling for BMI also revealed no statistical difference.

**Conclusions:**

Unlike the data presented in an animal model, no difference was demonstrated in the duration and quantity of serosanguinous drainage related to the use of MPH in patients undergoing mastectomy for the treatment of breast cancer. BMI greater than 30 was identified as an independent risk factor for high drain output and this risk was not affected by MPH use. NCT03647930, retrospectively registered 08/2018.

## Background

The most common complication following breast cancer (BC) surgery is seroma formation [1]. Reported incidence ranges from 15 to 90% [1, 2]. A seroma results from an accumulation of serous fluid in the dead space of the breast, under the skin flaps, or axilla following breast surgery. Although the exact pathogenesis of seroma formation is still unknown, accumulation of acute inflammatory exudate in response to surgical trauma during a prolonged acute phase of healing is thought to play a key factor [3, 4]. Several surgical techniques have been used to reduce seroma formation; use of ultrasonic scissors, physical closure of dead space, suction drainage, and placement of external compression dressings [3, 5]. Attempted chemical obliterations of dead space have also been assessed with mixed results [3, 5–8]. To date, no method has been described to consistently and reliably prevent seroma formation.

Microporous polysaccharide hemospheres (MPH) are hydrophilic polysaccharide particles (diameter of 30–100 μm) prepared from 100% purified potato starch and currently used as an absorbable hemostatic agent [9–11]. MPH is fully absorbed and enzymatically cleared from the wound within 24 to 48 h [9, 10]. Plant based polysaccharides have been shown to play a positive role in immune stimulation and wound healing via macrophage activation, fibroblast stimulation, and T-cell stimulation [12–17]. In addition to its immunostimulatory effects, MPH particles extract fluid from the blood, swell, and form a gelled matrix concentrating serum proteins, platelets, albumin, thrombin, and fibrinogen; creating a scaffold for the formation of fibrin clot [9, 18]. These characteristics may also help MPH prevent vascular and lymphatic drainage and prevent seroma formation.

An animal model evaluating topical MPH and seroma formation after mastectomy with axillary dissection showed a significant decrease in seroma volume [18]. Additionally, they demonstrated a reduction in the seroma’s total protein level, albumin concentration, lactate dehydrogenase level, and white blood cell counts; indicating a reduction in the accumulation of inflammatory exudate [18]. Histopathological evaluation demonstrated that the MPH group had decreased fibrous tissue and decreased number of macrophages and fibroblasts compared to the control; interpretation made was that MPH reduced seroma formation by accelerating the wound healing process [18].

We hypothesize that the unique immunostimulatory and hemostatic characteristics of MPH will have a positive role in the acceleration of wound healing decreasing the accumulation of acute inflammatory exudate and prevention of capillary and lymphatic leakage; therefore, decrease duration and quantity of serosanguinous drainage. The purpose of this study was to evaluate the efficacy of topical MPH used together with closed suction drainage, compared to suction drainage alone, in the reduction of drain output and time to drain removal following mastectomy for the treatment of BC. Previous studies relate the risk of seroma formation to high drain output prior to removal and early drain removal [19–26], therefore, high and/or prolonged drain output will be used as an indication of an increased risk of seroma formation.

## Methods

Prospective randomized single-blinded clinical trial of patients undergoing mastectomy for the treatment of BC conducted at a single center with a specialized breast center. Inclusion criteria were patient age ≥ 18 years undergoing simple mastectomy (SM) with or without sentinel lymph node biopsy (SLNB) or modified radical mastectomy (MRM) for the treatment of BC. Exclusion criteria were patients undergoing partial mastectomy, sentinel node biopsy requiring conversion to axillary lymph node dissection (ALND), immediate reconstructive surgery, systemic anticoagulation, or those choosing not to participate. Patients on antiplatelet therapies were not excluded from participation in this study. However, all platelet inhibitors, except for aspirin, were held for 7 days prior to surgery and resumed after the drains were removed. Study was approved by the Institutional Review Board at Memorial Health University Medical Center. Written informed consent was obtained by a surgery resident, breast center office nurse or research nurse during preoperative clinic visits or by the attending surgeon or surgery resident in the preoperative holding.

Between June 2012 and June 2014, fifty patients were enrolled into the study. The surgeon was blinded to patient enrollment during preoperative planning. Primary endpoints were time to drain removal, total drain output, and first 72-h drain output. Secondary endpoints were daily drain output, number of clinic visits, and postoperative complications. Medafor, Hemostatic Polymer Technologies Inc., Minneapolis, MN, provided the product MPH (Arista™ AH) at no cost to the patient or institution; this was included in the informed consent document.

Patient randomization, MPH vs. no-MPH, was performed via a random number generator program with two variables. The randomization list was generated by Randomization Generator, Medical Statistics Research Unit, University of Southampton, Southampton, UK. The principal investigator received a packet of 70 sealed opaque envelopes numbered in sequence containing a group assignment card. Randomization scheme utilized an equal allocation algorithm to ensure equal sample sizes at the conclusion of patient accrual. The act of randomization occurred when the patient entered the operating room. At that time, the surgeon opened the sealed envelope and read the group assignment card. For patients who underwent bilateral mastectomy, each breast was individually randomized and the surgeon opened separate assignment envelopes for the left and right breast. Patients were blinded to their randomization group throughout the course of the study.

All operations were supervised or performed by the same attending surgeon. All patients received pre-operative antibiotic prophylaxis administered within sixty minutes prior to surgical incision. Control of hemostasis was performed with knot-tying ligation and electrocautery. Following completion of the surgical procedure and randomization, the treatment group had a fixed five gram dose of MPH locally applied to the chest wall, skin flaps, and axillary wound if present. No application was performed in the control group. In all patients, the wound was closed over a closed suction drain under the skin flaps introduced through the lower flap in the axillary region; a second drain was placed in the axilla if ALND was performed. A light dressing was applied for 24 h. Patients were admitted to the hospital for observation and pain control and discharged home on postoperative day (POD) one with their drains. No post-operative antibiotic prophylaxis was given.

Prior to discharge, patients were instructed on how to measure, record, and discard the drainage. Patients were asked to call the clinic daily and report how much drainage was discarded. Follow-up was done in the outpatient breast center and drains were pulled when output was ≤ 25 ml per 24-h period or when no longer functional. In patients who underwent an ALND, only the output and management of the drain under the mastectomy skin flap was included in this analysis. The drain under the mastectomy skin flap was either removed first or at the same time as the axillary drain if both drains meet criteria for removal. Prospectively data collection of age, body mass index (BMI), procedure performed, menopausal status, tumor pathology and TNM stage, and primary and secondary endpoints was performed.

Prior to initiation of the study, power analysis indicated that group sample sizes of 32 (64 total patients) were needed to achieve 91% power to detect a difference between the two groups with a significance level of 0.05 using a two-sided two-sample independent t-test. However, during the time of the study Medafor, Hemostatic Polymer Technologies Inc. obtained new ownership and no longer wished to sponsor the study. At this time the trial had a total of 50 patients enrolled. Re-evaluation of the study was performed using the same calculations as above with 50 patients and found the power to be 90%. Due to the loss of funding and that the study was still considered to have enough power, the trial was terminated. Descriptive and inferential analyses were performed via the IBM Statistical Package for the Social Sciences software (IBM Corp. Released 2013. IBM SPSS Statistics for Windows, Version 22.0. Armonk, NY: IBM Corp.). The level of statistical significance was set at 0.05. Differences between groups were assessed for significance via chi-square and independent-samples t-tests.

## Results

**Fig. 1:**
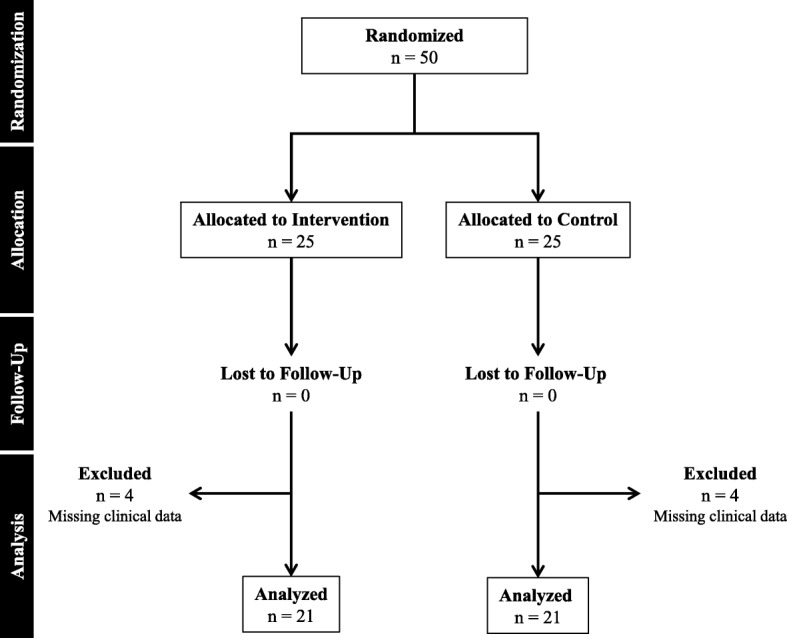
Flow diagram of the subjects who participated in the clinical trial

Patient recruitment and follow-up was performed between June 2012 to June 2014 with a total of 50 female patients enrolled and randomized. The clinical trial was terminated early due to loss of funding. All patients completed follow-up visits; however, 8 patients were excluded from analysis (four from each group) due to missing clinical data. The missing clinical data was due to either patient noncompliance with recording/reporting daily drain output, incomplete documentation of primary endpoints, or patient no longer wishing to participate in the study. A total of 42 patients were analyzed; 21 were in the MPH group and 21 were in the control group (Fig. 1). Characteristics of the study population were evaluated (Table 1). The mean post-operative time until drain removal was 9.6 (SD = 3.4, range 4–23) days, mean first 72-h drain output was 257.2 mL (SD = 80.9, range 130–505), and mean total drain output was 585.7 mL (SD = 353.2, range 149–2008). Only one patient experienced a postoperative complication; wound infection and dehiscence. This patient was in the MPH group and had a BMI < 30. No patient developed a residual or recurrent seroma after drain removal.

**Table 1:** Patient Characteristics

	Study Population	Control	vs.	MPH	*p*	BMI < 30	vs.	BMI ≥ 30	*p*
N	42	21		21		25		17	
Demographics									
Age (yrs.), mean ± SD (range)	64.9 ± 4.5 (23–89)	68.2 ± 15.7 (23–89)		61.2 ± 12.6 (34–82)	0.140	69.2 ± 13.59 (38–89)		58.6 ± 13.8 (23–80)	0.017
BMI, mean ± SD (range)	30.7 ± 8.3 (19–59)	30.9 ± 8.4 (19–59)		30.4 ± 8.4 (21–59)	0.843	26.2 ± 2.9 (19–30)		37.3 ± 9.3 (31–59)	0.000
Postmenopausal	92.9%	95.2%		90.5%	0.500	96.0%		88.2%	0.338
Procedure									
SM without SLNB	21.4%	55.6%		44.4%	0.605	20.0%		23.5%	0.784
SM with SLNB	47.6%	55.0%		45.0%		48.0%		47.1%	0.952
MRM	31%	38.5%		61.5%		32.0%		29.4%	0.859
Pathology									
Benign	14.3%	23.8%		4.8%	0.030	4.0%		20.0%	0.135
ADH	2.4%	4.8%		0%		4.0%		0%	
DCIS	14.3%	4.8%		23.8%		20.0%		4.0%	
IDC	59.5%	66.7%		52.4%		64.0%		36.0%	
ILC	9.5%	0%		9.4%		8.0%		8.0%	
Stage									
T 0	26.2%	23.7%		33.5%	0.130	24%		29.4%	0.807
T 1	31%	42.9%		19.0%		32%		29.4%	
T 2	26.2%	28.6%		30.8%		24%		29.4%	
T 3	11.9%	0%		11.9%		12%		11.8%	
T 4	4.8%	4.8%		4.8%		8%		0%	
N x	4.7%	4.80%		4.8%	.0755	4.00%		0.0%	0.729
N 0	52.4%	57.10%		52.4%		52.00%		44.0%	
N 1	16.7%	19.00%		16.7%		16.00%		12.0%	
N 2	11.9%	9.50%		11.9%		16.00%		4.0%	
N 3	14.3%	9.5%		14.3%		16.00%		8.0%	
Resection									
R0	97.4%	100%		95.2%	0.311	100%		94%	0.220
R1	2.4%	0.0%		4.8%		0%		6%	

*MPH* microporous polysaccharide hemospheres, *SD* standard deviation, *BMI* body mass index; SM: simple mastectomy, *SLNB* sentinel lymph node biopsy, *MRM* modified radical mastectom,; *ADH* atypical ductal hyperplasia, *DCIS* ductal carcinoma in situ, *IDC* invasive ductal carcinoma, *ILC* invasive lobular carcinoma

A comparison of the MPH and control groups’ patient characteristics was performed (Table 1). The only significant finding between the two groups was final pathology. Both groups had an equal distribution of benign and malignant disease; however the control group tended to have more benign findings while the MPH group had more DCIS. A comparison between MPH and control patients was made on all primary and secondary endpoints (Table 2). No significant difference was identified between the MPH and control groups. The impact of patient variables (age, menopausal status, BMI, procedure performed, tumor pathology, and TNM stage) on total number of drain days and total drain output were analyzed to determine their predictive value of high and/or prolonged drain output. Only BMI was identified as a significant predictor for high drain output.

**Table 2:** Outcomes Based on Microporous Polysaccharide Hemospheres Treatment and Body Mass Index

	Control	vs.	MPH	*p*	BMI < 30	vs.	BMI ≥ 30	*p*
Number of patients	21		21		25		17	
Number of post-op clinic visits	1.0		1.24	0.329	1.2		1.0	0.416
Post-op complication rate	0.0%		4.8%	0.500	4.0%		0.0%	0.416
Total drain days, mean ± SD (range)								
All procedures	10.0 ± 4.4 (4–23)		9.14 ± 2.9 5–16)	0.462	8.7 ± 2.8 (4–16)		10.9 ± 4.6 (6–23)	0.058
SM without SLNB	12.6 ± 6.5 (8–23)		9.8 ± 3.6 (7–15)	0.461	8.2 ± 0.8 (7–9)		15.3 ± 6.1 (8–23)	0.036
SM with SLNB	8.1 ± 3.1 (4–16)		8.2 ± 1.6 (5–11)	0.911	7.8 ± 2.1 (4–11)		8.6 ± 3.2 (6–16)	0.506
MRM	11.6 ± 2.9 (8–14)		9.9 ± 3.7 (6–16)	0.398	10.3 ± 3.8 6–16)		11 ± 2.9 (8–15)	0.717
Total drain output (mL), mean ± SD (range)								
All procedures	608.8 ± 409.7 (149–2008)		562.6 ± 294.6 (228–1289)	0.677	496.5 ± 244.9 (149–1290)		716.9 ± 446.1 (301–2008)	0.046
SM without SLNB	859.2 ± 675.9 (414–2008)		509.6 ± 299 (328–953)	0.372	391.8 ± 60.0 (328.0–459-0)		1093.9 ± 647 (482–2008)	0.044
SM with SLNB	434.2 ± 219.6 (149–996)		470.4 ± 126.4 (287–685)	0.666	432.4 ± 149.1 (149–685)		477.6 ± 227.5 (301–996)	0.596
MRM	742.7 ± 278.5 (432–1032)		692.7 ± 399.4 (228–1290)	0.812	658.1 ± 352.1 (228–1290)		798.1 ± 355.8 (432–1261)	0.502
1st 72-h output (mL), mean ± SD (range)								
All procedures	261.3 ± 88.3 (130–505)		253.2 ± =74.8 (155–403)	0.754	235.7 ± 66.9 (130–403)		288.7 ± 91.1 (177–505)	0.035
SM without SLNB	302.4 ± 118.2 (280–505)		198.4 ± 62.8 (155–201)	0.159	191.6 ± 37.4 (155–247)		336.9 ± 114.4 (249–505)	0.030
SM with SLNB	221 ± 59.7 (130–355)		244.1 ± 49.9 (177–330)	0.368	227.6 ± 55.4 (130–330)		237.1 ± 58.5 (177–353)	0.717
MRM	308.2 ± 82.5 (230–415)		290.8 ± 89.5 (167–403)	0.733	275.4 ± 80.0 (167–403)		332.8 ± 85.7 (221–415)	0.246

*SD* standard deviation, *SM* simple mastectomy; SLNB: sentinel lymph node biopsy, MRM: modified radical mastectomy

The sample population was split into groups based on BMI; BMI < 30 and BMI ≥ 30. A comparison of patient characteristics between the two groups was performed (Table 1); only significant findings were BMI and age (patients with BMI < 30 tended to be older). A post hoc analysis was performed and the two groups were analyzed for all primary and secondary endpoints (Table 2). The BMI ≥ 30 group had a significantly higher total drain output (716.9 mL vs. 496.5 mL, *p* = 0.046) and first 72-h drain output (288.7 mL vs. 235.7 mL, *p* = 0.035). The BMI ≥ 30 group also had significantly higher drain output on POD-1 (107.6 mL vs. 87.3 mL, *p* = 0.022), POD-2 (96.5 mL vs. 78.2 mL, *p* = 0.034), POD-10 (24.4 mL vs. 7.7 mL, *p* = 0.041), and POD-15 (9.6 mL vs. 1.1 mL, *p* = 0.046). Evaluation by procedure demonstrated a significant increase total number of drain days (15.3 vs. 8.2, *p* = 0.036), total drain output (1093.9 mL vs. 391.8 mL, *p* = 0.044), and first 72-h output (336.9 mL vs. 191.6 mL, *p* = 0.030) for patients undergoing a SM without SLNB with BMI ≥ 30.

A post hoc analysis of MPH vs. control was performed controlling for BMI < 30 and BMI ≥ 30. No significant difference was again identified between the MPH and control groups in evaluation of all the primary and secondary end-points.

## Discussion

This is the first clinical study evaluating topical MPH and the risk of seroma formation as measured by high and/or prolonged drain output in patients undergoing mastectomy for the treatment of BC. In designing the clinical trial, the hypothesis was made that immunostimulatory and hemostatic properties of MPH would aid in the prevention of postoperative inflammatory exudate accumulation and capillary and lymphatic leakage, thereby decreasing the risk of seroma formation as measure by time to drain removal and drain output. A total of 50 patients were evaluated in this prospective randomized trial. The results of this trial failed to show a significant decrease in drain duration or quantity of drainage with the use of MPH. This suggests that MPH’s immunostimulatory and hemostatic properties may be inadequate to decrease the risk of seroma formation.

Several studies have also assessed the use of other topical and systemic pharmaceutical agents to reduce seroma formation. Multiple studies have evaluated the use of fibrin glue with mixed results; some reporting no difference [7, 27–31] and others reporting a decrease in seroma formation [32, 33]. A study evaluating the use of topical thrombin failed to show a reduction in seroma formation [8]. Other studies have also evaluated the use of tetracycline, as a sclerosant, for the reduction of seroma formation with mixed results in its efficacy [34–36]. The use of systemic somatostatin analog treatments has also been evaluated, again with mixed results. A study evaluating octreotide demonstrated a decrease in drain output [37], while a study evaluating lanreotide did not [38]. The significant methodological, sample size and clinical diversity between all these studies make it difficult to generate an overall conclusion. Despite all these efforts, no single agent has been identified for optimal prevention of this complication.

In addition to the surgical disruption of lymphatics and the creation of dead space, accumulation of acute inflammatory exudate in response to surgical trauma and prolonged wound healing also plays a key role in seroma formation [3, 4]. Therefore, local wound immune stimulation and acceleration of the inflammatory stage of wound healing may help prevent seroma formation. OK-432 is a streptococcal preparation (made from a low-virulence Group A streptococcus) used as an immunostimulatory agent [39, 40]. A study evaluating OK-432 in seroma formation after ALND for BC demonstrated a significant reduction in postoperative drainage and drain duration [6]. The mechanism of reduced drainage with OK-432 was proposed to be through immune activation, cytokine release, and induction of an accelerated inflammatory response [6].

Some reported predisposing factors for seroma formation are age, obesity, breast size, presence and number of malignant axillary lymph nodes, previous breast biopsies, history of prior neoadjuvant chemotherapy, MRM, delayed breast reconstruction, BC stage, and the use of heparin or tamoxifen [1, 5, 41, 42]. In this clinical trial obesity was the only significant predictor of high drain output; defined by significantly higher mean total drain output and first 72-h drain output. Controlling for BMI, an evaluation of MPH in the risk of seroma formation (as measured by high or prolonged drain output) still demonstrated no significant difference related to the use of MPH. Patients included in this study underwent a mix of operative procedures; simple mastectomy without sentinel lymph node biopsy (21% of patients), simple mastectomy with sentinel lymph node biopsy (48% of patients), or modified radical mastectomy (31% of patients). Although MRM has been reported to be a predisposing factor for seroma formation, we did not appreciate a significant difference in total drain days or total drain output between the three procedure groups. However, this study was not powered to evaluate a difference in drain production between the operative procedures.

The overall wound complication rate in this study was 2.4%, with only one of 42 patients developing a wound complication. This is slightly lower than the overall wound complication rates for breast surgery reported in the literature, ranging from 5 to 9% [42–44]. In this study, no incidence of wound infection was seen. The postoperative wound infection reported in the literature range from 0 to 16%, with lower wound infection rates in patients who undergo ambulatory surgery (0–2%) [43, 44]. All the patients in this study were discharged on postoperative day one, with less than 24 h stay in the hospital. This may contribute to the observed low wound infection rate. Interestingly, no patient developed a residual or recurrent seroma after drain removal. Several studies have evaluated the relationship of time of drain removal and the risk of seroma formation, with early drain removal associated with higher rates of seroma formation [45–49]. Studies with late drain removal, the reported seroma incidence rate ranged from 0 to 29% [44, 45, 47–49]. In this study, the drains were pulled when output was ≤ 25 ml in 24-h period with average drain duration of 9.5 days. This prolonged period of drainage may be contributing to our low rates of residual or recurrent seroma formation after drain removal. However, given that our institution is a large tertiary referral center, it is possible that patients with clinically insignificant or small seromas were treated by their local physicians and not sent back to our institution for evaluation.

Drain placement was a requirement for enrollment in this clinical trial. Therefore, all patients had a drain placed. However, several studies that have evaluated the risk of seroma formation following mastectomy without drain placement reporting increased postoperative seroma formation and higher seroma volumes [46, 50, 51]. Additional studies have evaluated the risk of sermoa formation following mastectomy without drain placement with the use of other surgical or chemical techniques for obliterations of dead space with some encouraging results [46, 52, 53]. Although encouraging, some major limiting factors of these studies include small sample sizes, retrospective studies, and reports suggesting that surgical techniques to close dead space have poor cosmetic results and potential increased morbidity [5, 54–57]. There are a few clinical trials currently ongoing evaluating surgical dead space obliteration and seroma formation which should be able to provide more clarity on this issue [58, 59]. At this time, continued controversy exist in drain usage and drainage method with approach primarily determined by clinical experience and surgeon preference.

Even though on post hoc evaluation the associated effect size indicated a good level of practical significance, one of the limitations of this study is the early termination of the trial decreasing our statistical power. Another limitation of the study is that eight enrolled patients were excluded for missing clinical data. This 16% dropout rate is higher than our expected rate of 10%, but still lower than dropout rates of over 30% reported in other clinical trials [60]. The missing data and patient exclusions in this study may reduce the benefit provided by the randomization, as noncompliance and dropouts can occur non-randomly and analyzing the data excluding those patients could lead to biased results. However, the number of patients excluded were split evenly across the treatment groups, potentially limiting the negative impact on the sample randomization. Additionally, the reduction in the patient sample size from exclusion of these patients slightly decreases our statistical power and data for this patients could have impacted our results. A third limitation of this study is that the wound healing process was not monitored and evaluation of the direct effect of MPH immune stimulation and reduction inflammatory exudate cannot be made. Although we did not identify a significant difference in the risk of seroma formation as measured by high or prolonged drain output with the use of MPH, a larger clinical trial would be needed to fully evaluate the immunostimulatory effects of MPH on inflammatory exudate accumulation and wound healing and potential correlation with seroma formation.

## Conclusion

A total of 50 patients were evaluated in this prospective randomized trial evaluating the effects of topical MPH on the risk of seroma formation following mastectomy for the treatment of BC as measured by prolonged total drain days and high drain output. Unlike the results presented in an animal model, this trial failed to show a significant reduction in the duration and quantity of serosanguinous drainage with the use of MPH. However, on multivariate analysis BMI ≥ 30 was identified as an independent risk factor for high drain output, which is indicative of a risk for postmastectomy seroma formation.

## Abbreviations

ALND: axillary lymph node dissection
BC: breast cancer
BMI: body mass index
MPH: microporous polysaccharide hemospheres
MRM: modified radical mastectomy
SLNB: sentinel lymph node biopsy
SM: simple mastectomy
